# Sex Differences in Outcomes After Type A Acute Aortic Dissection: An Analysis of the UK National Adult Cardiac Surgery Audit Registry

**DOI:** 10.3390/jcdd13090416

**Published:** 2026-08-26

**Authors:** Marco Gemelli, Thanakorn Rojanathagoon, Ettorino Di Tommaso, Maria Comanici, Eltayeb Mohamed Ahmed, Cha Rajakaruna, Gianni D. Angelini, Daniel P. Fudulu

**Affiliations:** Department of Cardiac Surgery, Bristol Heart Institute, University of Bristol, Bristol BS2 8ED, UK; m.gemelli@bristol.ac.uk (M.G.); fq18031@bristol.ac.uk (E.D.T.); maria.comanici@bristol.ac.uk (M.C.); eltayeb.mohamedahmed@uhbw.nhs.uk (E.M.A.); cha.rajakaruna@uhbw.nhs.uk (C.R.); g.d.angelini@bristol.ac.uk (G.D.A.)

**Keywords:** aortic dissection, sex, outcomes, NACSA

## Abstract

(1) Background: Type A acute aortic dissection (TAAAD) predominantly affects men, yet conflicting evidence persists regarding sex- and socioeconomic-related differences in outcomes. We evaluated these differences using a large national UK registry. (2) Methods: Patients undergoing TAAAD repair between 1996 and 2019 were identified from the National Adult Cardiac Surgery Audit (NACSA) and stratified by sex and socioeconomic status. In-hospital outcomes were compared and inverse probability weighting was applied to adjust for imbalances between groups and estimate the average treatment effect (ATE) of female versus male. (3) Results: A total of 2857 patients underwent TAAAD repair, including 1907 (67%) men and 950 (33%) women. Women were older and had lower BMI, while men more frequently presented with bicuspid aortic valve and aortic root aneurysm. The rates of aortic root replacement and total arch replacement were comparable between sexes, as was the median circulatory arrest time. No significant differences were observed in socioeconomic deprivation indices. In-hospital mortality (18.7% in men vs. 19% in women), cerebrovascular accident (12.5% vs. 12.5%), dialysis (14.5% vs. 14.6%), and length of stay (13 vs. 13 days) were comparable between sexes. After inverse probability weighting, female sex was not independently associated with in-hospital mortality or post-operative complications. (4) Conclusions: In this large UK registry, female sex was not independently associated with worse in-hospital outcomes after TAAAD repair.

## 1. Introduction

Type A acute aortic dissection (TAAAD) is a life-threatening condition requiring emergency surgery and remains associated with substantial morbidity and mortality [1,2]. Although TAAAD predominantly affects men, the impact of sex on surgical outcomes remains controversial. Early reports from the International Registry of Acute Aortic Dissection demonstrated higher operative mortality in women; however, more recent IRAD cohorts suggest that female sex is no longer an independent predictor of adverse outcomes [3,4]. Other studies have reported conflicting findings. A recent large meta-analysis including more than 16,000 patients demonstrated no significant sex-related differences in in-hospital mortality, although long-term survival appeared to be more favourable in male patients [5]. Despite these findings, sex-based disparities in aortic surgery outcomes remain an important knowledge gap, as highlighted in contemporary American Heart Association guidelines [6].

Using data from a large national registry, this study aimed to evaluate the real-world data impact of sex on post-operative outcomes following surgical repair of TAAAD.

## 2. Materials and Methods

The National Adult Cardiac Surgery Audit (NACSA) is a mandatory, prospectively collected registry that reflects real-world practice across the UK National Health Service and captures data on all major adult cardiac surgical procedures performed throughout the United Kingdom [7]. The Health Research Authority and Health and Care Research Wales approved the study and a waiver for patients’ consent was obtained. This study was conducted in accordance with the Declaration of Helsinki. Patients undergoing surgical repair for Type A acute aortic dissection between 1999 and 2019 were identified and stratified by sex and socioeconomic status. Clinical characteristics, socioeconomic factors, and in-hospital outcomes were analysed accordingly. Socioeconomic status was assessed using the Index of Multiple Deprivation (IMD) 2019 (Supplementary Table S1), a validated measure of relative deprivation at the small-area level based on Lower-layer Super Output Areas (LSOAs) in England [8].

Continuous variables are reported as mean ± standard deviation and categorical variables as counts and percentages; all tests were two-sided with *p* < 0.05 considered statistically significant. Missing data were handled by case-wise exclusion. There was no missing data in the matching variables included in the analysis, likely because these represent mandatory fields within the NACSA registry. Missingness in the outcome variables was 8.7% for postoperative dialysis, 0.6% for postop length of stay and 0.3% for in-hospital mortality. Female sex was treated as the exposure of interest, with male sex as the reference group, and inverse probability weighting (IPW) was applied to account for baseline differences between groups. IPW was used to balance measured baseline covariates between sexes and estimate the average treatment effect in the overall population while retaining the full study cohort, thereby avoiding exclusion of patients that typically occurs with propensity score matching (Supplementary Tables S2 and S3). Propensity scores were estimated using multivariable logistic regression including the following prespecified variables available before surgery: age, Index of Multiple Deprivation, hypertension, insulin-treated diabetes, peripheral vascular disease, pulmonary disease, serum creatinine > 200 μmol/L, neurological dysfunction, preoperative atrial fibrillation, recent myocardial infarction, NYHA class IV, severe left ventricular dysfunction, pulmonary hypertension, emergency status, Marfan syndrome, bicuspid aortic valve, aortic root aneurysm, thoracic aortic aneurysm, and year of surgery, and IPW weights were used to estimate the average treatment effect in the overall population. Because the study period spanned more than two decades, during which surgical technique, perioperative management, and case selection evolved, year of surgery was included in the propensity score model to adjust for secular trends in practice. As case volume increased substantially over the study period, potentially due to increased reporting, the year of surgery was modelled as a continuous variable using a restricted cubic spline (three degrees of freedom) rather than divided into fixed calendar-length epochs, avoiding unstable strata that a fixed-epoch categorical approach would have produced in the earliest years. Covariate balance before and after weighting was assessed using standardized mean differences (SMDs), with values <0.10 indicating adequate balance. Outcome comparisons were conducted in the weighted pseudo-population using weighted regression models: binary outcomes (mortality, transient ischemic attack, cerebrovascular accident, and postoperative dialysis) were analysed with weighted logistic regression and are reported as odds ratios with 95% confidence intervals, while postoperative length of stay was analysed using weighted linear regression and reported as mean differences with 95% confidence intervals. All analyses were performed using R (RStudio) and Quarto with the ipw, survey, cobalt, gtsummary, ggplot2, and tidyverse packages. The R statistical code for the propensity score model was iteratively refined with the assistance of an AI language model (Claude, Anthropic 3.7 Sonnet, Anthropic PBC, San Francisco, CA, USA); all code was reviewed, validated, and executed by the authors, who take full responsibility for the analysis.

## 3. Results

Among 677,323 patients who underwent cardiac surgery during the study period, 2857 (0.4%) underwent repair for Type A acute aortic dissection. The cohort comprised 1907 men (66.7%) and 950 women (33.3%). Women were older and had a lower prevalence of peripheral vascular disease and a higher prevalence of pulmonary disease. In contrast, men more frequently presented with bicuspid aortic valve and aortic root aneurysm. Baseline characteristics, operative details, and postoperative outcomes are summarised in Table 1.

The rates of aortic root replacement (22.8% in men vs. 18.8% in women; *p* = 0.2) and total arch replacement (12.4% vs. 12.5%; *p* > 0.9) were comparable between sexes, as was the mean circulatory arrest time (36 vs. 34 min, *p* = 0.8). No significant differences were observed in socioeconomic deprivation indices (*p* > 0.9).

In-hospital mortality was comparable between men and women (18.7% vs. 19.0%, *p* = 0.8). Similarly, no significant sex-related differences were observed in major postoperative outcomes, including cerebrovascular accident with full recovery (3.7% vs. 3.6%, *p* = 0.8), cerebrovascular accident with residual neurological deficit (8.8% vs. 8.9%, *p* = 0.9), new requirement for dialysis (14.5% vs. 14.6%, *p* = 0.8), and total length of hospital stay (median 13 days in both groups; *p* = 0.5).

After inverse probability weighting, female sex was not independently associated with in-hospital mortality compared with male sex (OR 0.93, 95% CI 0.71–1.2; *p* = 0.561). No significant associations were observed for transient ischemic attack (OR 0.71, 95% CI 0.41–1.24; *p* = 0.233), cerebrovascular accident (OR 0.86, 95% CI 0.61–1.21; *p* = 0.378), or postoperative dialysis (OR 1.07, 95% CI 0.81–1.41; *p* = 0.652). Female sex was also not associated with postoperative length of stay (mean difference 0.34 days, 95% CI −2.02 to 2.71; *p* = 0.777).

## 4. Discussion

In this large analysis of a nationwide UK registry, sex was not independently associated with worse in-hospital outcomes after surgical repair of Type A acute aortic dissection. These findings contribute to the ongoing debate on sex-related disparities following TAAAD repair. While early international registry data suggested higher operative mortality among women, more recent evidence indicates that sex is not an independent risk factor once age, comorbidities, and operative variables are considered [9,10]. Women undergoing TAAAD surgery were older and had a higher prevalence of pulmonary disease, whereas men more frequently presented with a bicuspid aortic valve and aortic root aneurysm. Importantly, operative management (including rates of aortic root and arch replacement and circulatory arrest times) was similar between sexes, potentially explaining the absence of outcome differences. No significant differences were observed in socioeconomic deprivation indices between men and women. Although socioeconomic status is an important determinant of cardiovascular outcomes, comparable deprivation profiles suggest it did not contribute to early sex-based disparities in this cohort [11].

The strengths of this study include the use of a large, mandatory national registry with prospective data collection and the application of robust statistical methods to mitigate confounding. Nevertheless, several limitations warrant consideration. The observational design precludes causal inference, and residual confounding cannot be entirely excluded. Unmeasured clinical and gender-related factors not captured in the registry may have influenced the results. Moreover, the analysis was limited to in-hospital outcomes, with no available data on long-term survival and rate of reintervention, disease presentation, or timing of surgical intervention.

## 5. Conclusions

In conclusion, female sex was not independently associated with worse in-hospital outcomes after adjustment for measured baseline characteristics, supporting the growing body of evidence suggesting comparable early surgical outcomes between sexes in contemporary practice. However, given the observational design and the potential influence of unmeasured or mediating factors, these findings should be interpreted with appropriate caution.

## Figures and Tables

**Table 1:** Baseline characteristics, details of aortic pathology, operative details and post-operative outcomes of the TAAAD population by sex. EF: ejection fraction. IPW: inverse-probability weighting. LV: left ventricle. NYHA: New York Heart Association. SD: standard deviation.

Patient Characteristics	Before IPW				After IPW (Weighted)		
	Male	Female	*p*-Value	SMD	Male	Female	SMD
Baseline characteristics							
Age, years, mean (SD)	61 (13)	67 (12)	<0.001	−0.47	62 (13)	62 (14)	0.02
Hypertension	1286 (67.4%)	673 (70.8%)	0.065	0.07	1243 (70.7%)	1243 (70.4%)	0.01
Diabetes on Insulin	7 (0.4%)	8 (0.8%)	0.11	0.06	11 (0.6%)	10 (0.6%)	0.01
Peripheral Vascular Disease	371 (19.5%)	152 (16.0%)	0.024	0.09	321 (18.3%)	346 (19.6%)	0.03
Pulmonary Disease	149 (7.8%)	120 (12.6%)	<0.001	0.16	172 (9.8%)	172 (9.7%)	0.00
Creatinine > 200 μmol/L	77 (4.0%)	28 (2.9%)	0.14	0.06	64 (3.6%)	64 (3.6%)	0.00
Neurological dysfunction	142 (7.4%)	74 (7.8%)	0.7	0.01	139 (7.9%)	152 (8.6%)	0.03
Preoperative atrial fibrillation	128 (6.7%)	69 (7.3%)	0.6	0.02	109 (6.2%)	109 (6.2%)	0.00
Recent myocardial infarction	104 (5.5%)	40 (4.2%)	0.2	0.06	88 (5.0%)	93 (5.3%)	0.01
NYHA Class IV	296 (15.5%)	131 (13.8%)	0.2	0.05	281 (16.0%)	284 (16.1%)	0.00
Poor LV Function (EF < 30%)	55 (2.9%)	26 (2.7%)	0.8	0.01	49 (2.8%)	54 (3.0%)	0.01
Pulmonary Hypertension	8 (0.4%)	4 (0.4%)	>0.9	0.00	8 (0.5%)	6 (0.3%)	0.02
Emergency procedure	1550 (81.3%)	759 (79.9%)	0.4	0.04	1432 (81.5%)	1442 (81.6%)	0.00

Aortic Pathology							
Marfan Syndrome	50 (2.6%)	28 (2.9%)	0.6	0.02	44 (2.5%)	44 (2.5%)	0.00
Bicuspid Aortic Valve	69 (3.6%)	23 (2.4%)	0.088	0.07	55 (3.2%)	65 (3.7%)	0.03
Aortic root aneurysm	100 (5.2%)	36 (3.8%)	0.085	0.07	52 (2.9%)	57 (3.2%)	0.02
Thoracic aortic aneurysm	215 (11.3%)	98 (10.3%)	0.4	0.03	124 (7.1%)	125 (7.1%)	0.00

Operative Details							
Aortic Root Replacement	435 (22.8%)	179 (18.8%)	0.2	0.10	373 (21.2%)	376 (21.3%)	0.00
Total Arch Replacement	237 (12.4%)	119 (12.5%)	>0.9	0.00	275 (15.7%)	273 (15.5%)	0.00
Circulatory Arrest Time, mean (SD)	36 (25)	34 (22)	0.8	0.05	35 (24)	35 (23)	0.00
							
Operative Year	2014.96 (2.60)	2015.15 (2.43)	−0.07	0.8	2015.21 (2.53)	2015.23 (2.49)	−0.01

## Data Availability

The data underlying this study were obtained from the registry and are not publicly available due to data governance and confidentiality restrictions. All data generated or analysed that are necessary to support the findings of this study are reported within the article and its Supplementary Materials. Additional aggregated or de-identified data may be available from the corresponding author upon reasonable request, subject to approval by the registry governance body and applicable data-sharing regulations.

## Supplementary Materials

The following supporting information can be downloaded at: https://www.mdpi.com/article/10.3390/jcdd13090416/s1, Table S1: Decile of the index of multiple deprivation in UK; Table S2: Missingness in Propensity Score Matching Variables; Table S3: Missingness in Outcome Variables.

## Abbreviations

IMD	Index of Multiple Deprivation
IPW	Inverse Probability Weighting
NACSA	National Adult Cardiac Surgery Audit
TAAAD	Type A Acute Aortic Dissection
