# Supplementary material for: Sex Differences in Outcomes After Type A Acute Aortic Dissection: An Analysis of the UK National Adult Cardiac Surgery Audit Registry
Source: J Cardiovasc Dev Dis. 2026 Aug 26;13(9):416. doi: 10.3390/jcdd13090416 (PMC13607030; doi:10.3390/jcdd13090416)
Supplement: Supplementary file 1 [file jcdd-13-00416-s001.zip › jcdd-4422134-supplementary.pdf]

Supplementary Materials

Supplementary Table S1: Decile of the index of multiple deprivation in UK

Decile of the index of multiple deprivation in UK	Male N = 1,907	Female N = 950	P-value
Most deprived decile	189 (11%)	94 (11%)	>0.9
2nd decile	166 (10%)	73 (8.8%)	>0.9
3rd decile	155 (9.4%)	80 (9.7%)	>0.9
4th decile	151 (9.1%)	62 (7.5%)	>0.9
5th decile	163 (9.8%)	73 (8.8%)	>0.9
6th decile	157 (9.5%)	89 (11%)	>0.9
7th decile	165 (10.0%)	76 (9.2%)	>0.9
8th decile	154 (9.3%)	101 (12%)	0.2
9th decile	177 (11%)	93 (11%)	>0.9
Least deprived decile	180 (11%)	85 (10%)	>0.9

Supplementary Table S2: Missingness in Propensity Score Matching Variables

Missingness in Propensity Score Matching Variables.

Variable	N Missing	N Total	% Missing
CircArrest	1100	2857	38.5
Gender	0	2857	0.0
Age	0	2857	0.0
Hypertension	0	2857	0.0
NeuroDys	0	2857	0.0
Cr200	0	2857	0.0
RecentMI	0	2857	0.0
PulmonaryDisease	0	2857	0.0

Missingness in Propensity Score Matching Variables.

Variable	N Missing	N Total	% Missing
NYHA4	0	2857	0.0
PulHTN	0	2857	0.0
Diabetes.Insulin	0	2857	0.0
LVF1.Poor	0	2857	0.0
PVD	0	2857	0.0
PreopAF	0	2857	0.0
Urgency.Emergency	0	2857	0.0
Ao.Aetiology.Marfan	0	2857	0.0
Ao.Root.Path.Aneurysm	0	2857	0.0
Ao.Aetiology.Bicuspid	0	2857	0.0
thoracic_aneursym	0	2857	0.0
root	0	2857	0.0
total_arch	0	2857	0.0
OpYear_c	0	2857	0.0

Supplementary Table S3: Missingness in Outcome Variables.

Outcome	N Missing	N Total	% Missing
PostopDialysis	153	1757	8.7
PostLOS	10	1757	0.6
Mortality	5	1757	0.3
TIA	0	1757	0.0
CVA	0	1757	0.0
